# Contextual determinants of multiple sexual partnerships amongst young people in South Africa: a multilevel analysis

**DOI:** 10.1186/s12889-024-18872-5

**Published:** 2024-06-07

**Authors:** Nebechukwu H. Ugwu, Clifford O. Odimegwu

**Affiliations:** 1 Demography and Population Studies Programme, Schools of Public Health and Social Sciences, University of the Witwatersrand, Johannesburg, South Africa; 2 Institute for Development Studies, University of Nigeria, Enugu Campus, Enugu, Nigeria

**Keywords:** Contextual factors, Multiple sexual partnerships, Young people, South Africa

## Abstract

**Background:**

Risky sexual behaviour (RSB), particularly multiple sexual partnerships (MSP) continues to be a major public health concern and has been linked to the increasing STIs, including HIV/AIDS in many parts of sub-Saharan Africa (SSA), suggesting that there is an association between contextual factors and multiple sexual partnering. However, in South Africa, this association is not well established in recent literature. Hence, this study examined the contextual factors contributing to multiple sexual partnerships among young people in South Africa.

**Materials and methods:**

Data was extracted from the 2016 South Africa Demographics and Health Survey (2016 SADHS). A cross-sectional study of 3889 never-married young people. Descriptive and inferential statistics as well as multilevel logistic regression were used to analyse the data on never-married young people aged 15 to 24 years.

**Results:**

The results indicated that at the individual level, young males (61.7%) were significantly more likely than their female counterparts (56.1%) to engage in multiple sexual partners, although, the difference was not as significant as expected. At the community level clustering, the likelihood of exposure to multiple sexual partnerships significantly increased among females (OR = 1.47; 95% CI: 1.25–1.73) but decreased among their male counterparts (OR = 0.73; 95% CI: 0.58–0.92), in particular, family disruption, residential instability, and ethnic diversity led young people to engage in multiple sexual partnerships.

**Conclusions:**

There is a need to intensify programmes aimed at considering appropriate policy options to reduce the prevalence of multiple sexual partnerships. Adopting the implications of these findings is essential for a developmental strategy towards achieving the sustainable development goal of ending STIs among young people in South Africa.

## Introduction

Globally, youth engagement in risky sexual behaviour continues to be a major public health concern and is linked to the increasing sexually transmitted infections (STIs), including HIV/AIDS among young people [1]. Despite many international, national, and sub-national efforts to reduce risky sexual behaviour (RSB) among young people, especially in sub-Saharan Africa, the negative effects it has on the health and well-being of young people continue to widen [2]. Any sexual activity with two or more people within a specific time is defined as multiple sexual partnerships [2, 3]. The risks of MSP, particularly those without the use of contraceptives, include exposure to STIs, including HIV/AIDS, unwanted pregnancies, maternal morbidity due to pregnancy complications and unsafe abortions [3–6]. These risks can harm young people’s health and quality of life. For instance, over half of all abortions in SSA are performed on adolescents and young women under the age of 25 [7].

The government of South Africa has made efforts at the national level to establish the Adolescent and Youth Policy and the National Strategic Framework for HIV/AIDS, STIs, and TB 2012–2016, which are vital national policies in South Africa geared towards addressing youth health, sexuality, and sexual and reproductive health (SRH). Furthermore, high levels of HIV education and condom use have intensified. However, evidence shows that there are persistent engagements of youth in (RSB) such as MSPs in South Africa, necessitating for further investigation into the factors responsible for this high rise of RSB among the youth. Moreover, there has been an increase in the prevalence of RSB among young people in South Africa [Stat SA]. For instance, the prevalence of risky sexual behaviours such as MSPs has been found to range between 18.3% in 2012 [6] and 23.5% in 2021 [8], which is higher than Uganda estimates of 2.2-6.6% for males and females respectively [9]. Despite that, the two countries have almost the same population, with South Africa slightly higher. Furthermore, only 21% of 15-19-year-olds and 17% of 20-24-year-olds used condoms during their most recent sexual engagement in 2018, and more than 41.1% of youth reported more than two sexual partners [10]. Statistics reported that economic hardships contribute to an increase in RSB among young people. For instance, it was reported that 55.5% of households in South Africa were living below the national poverty level. Also, about four out of 10 families (39.8%) were headed by women in 2019 [11]. This may be attributed to 20,980 divorces recorded in 2020 across South Africa, with Cape Town having the largest proportion at 23.8% [11]. There is also an increasing unemployment rate, with 32.4% of youth aged between 15 and 24 years not employed, educated, or trained [11]. This is evidence that community factors play a vital role in youth engagement in RSB.

Previous studies on RSB conducted in different countries in SSA, including South Africa have identified individual and household-level-related factors, such as gender [12], age [13], education attainment [14], employment status [15], and household size [12], are responsible for an increase in RSB among youth, whereas less attention has been paid to contextual factors. Both Murudi [16] and Seff et al. [17] found contextual factors such as neighbourhood poverty, residential mobility, family disruption, and ethnic/racial heterogeneity as being associated with RSB. Adeyemo and Williams [18] found that the absence of both parents is associated with MSPs. It is important to investigate these contextual determinants of RSB, especially in a country such as South Africa, which often experiences these issues among its youth, to understand and tackle the underlying contextual pathway of RSB on youth sexual behaviour.

However, there are no known studies conducted in South Africa to examine the influence of contextual factors contributing to youths’ engagement in MSPs using national data. Investigating contextual determinants of MSPs would be key to informing the designing of evidence-based interventions to address the sexual needs of this young population. Therefore, the results of this current study will help policymakers implement context-specific interventions aimed at reducing risky sexual practices among young people in the country. The study findings will also provide evidence to give direction for multisectoral agencies to intensify their strategies to reduce RSB and its associated consequences. Therefore, this study aims to examine the critical factors of individual and contextual factors that are associated with MSPs among youths in South Africa using a lens of social disorganisation theory.

## Theoretical frameworks

This study used the Social Disorganisation Theory (SDT) developed by Shaw and McKay [19] to examine the sexual risk behaviours of South African youth [20]. The theory was developed to explain variations in crime and delinquency among adolescents in the inner-city neighbourhoods of Chicago, Illinois, USA. Social disorganisation identifies neighbourhood poverty, residential instability due to migration, family dysfunction/disruption and racial and ethnic heterogeneity as the main structural components that lower the capability of communities to regulate themselves, including youth activities [21, 22]. The SDT argues that these structural features in certain neighbourhoods diminish community attachments, social norms, and social ties by weakening community-level social control of crime and deviance [21, 23, 24]. Second, the SDT proposes that neighbourhoods characterised by a high level of these structural disadvantages are socially disorganised and therefore unable to regulate themselves and cannot socialise the residents, especially adolescents, to engage in conventional behaviours. Although the SDT was originally applied to crime and delinquency, researchers such as Odimegwu and Ugwu [12] and Opoku-Ware et al. [25] have applied the theory to different behavioural studies such as youth sexual risk behaviours, sexual and gender-based violence, rape, teenage pregnancy, and educational behaviour among adolescents.

The SDT frameworks have been identified as suitable for this study because they suggest that high-risk sexual behaviour is multifaceted phenomenon grounded in the interplay of individual, family, community, and social factors. These factors include age, education, employment status, neighbourhood poverty, residential instability, family disruption (female-headed households), household size, and proximity to urban areas as key structural factors that diminish individual-level and community-level self-regulatory capacities [19]. One report indicates that social disorganisation theory is employed to explain at a high level how individual-level (age, educational status, employment status) and community-level factors (poverty, family disruption, residential instability, race/ethnic disparities), directly or indirectly influence the reproductive health of residents of a particular neighbourhood including young people’s sexual behaviour [26]. However, very few studies have examined how both individual and community level factors are associated with multiple sexual activities in adolescents in South Africa. Hence, this study examined the socio-ecological determinants of MSP among young people in South Africa, with an emphasis on the role of social disorganisation.

## Materials and methods

### Study area and population

This study utilised the most recent secondary data from the 2016 South Africa Demographics and Health Survey (SADHS) dataset based on availability. The SADHS 2016 is a cross-sectional study conducted by Statistics South Africa [27] in partnership with the South African Medical Research Council (SAMRC) at the request of the National Department of Health (NDoH). The survey used of the sampling frame from the Statistics South Africa Master Sample Frame (MSF), which was created using census 2011 enumeration areas (EAs). Due to the geographical hierarchy structure of the census, which links enumeration areas to administrative boundaries, information is available at municipal, district, and provincial levels in the survey. The survey provides up-to-date information from males and females to the National Department of health and policymakers on demographic and health indicators. The details of the research design and methodology used in the survey are well described in the full report [27]. For this study, the analyses covered a weighted sample of 3,889 young people (females − 2,621 and males − 1,268) who reported not being married during the survey. The analysis was based on data from ‘young people’ defined as those aged 15–24 years, which conforms to the World Health Organisation (WHO) definition, and the term is used interchangeably with ‘adolescents’ and ‘youth’ [28].

### Outcome variables

Information on multiple sexual partners is vital in preventing sexually transmitted infections, including HIV, and monitoring intervention programmes to control the spread of the epidemic, especially among never-married young people. To assess if participants had MSP, all eligible young people were asked the following question: “how many sexual partners, excluding the spouse, do you have?” The two possible outcomes for the questions were “1” meaning a single partner or “2 +” meaning having two or more partners in the survey period. Respondents who reported having more than one sexual partner were coded “1” while those who reported otherwise formed the other group of the dichotomy and were coded “0”. All the young people who did not respond to the question were excluded from the study. In this study, a 12-month reference period was used to capture the most recent sexual partnerships and minimise recall errors [29]. The focus was on the number of sexual partners in the last 12 months that preceded the survey because having a sexual partner constitutes one of the key pathways through which young people can be exposed to STIs, including HIV/AIDS. See Table 1 for details.

### Explanatory variables

The key explanatory variables were neighbourhood poverty, residential mobility, family disruption, and ethnic/racial diversity. Neighbourhood poverty was measured as the percentage of households in the poorest quintile of the wealth index [30]. Residential mobility is defined as the proportion of households occupied by persons who had moved from another dwelling during the previous 5 years [31, 32]. Family disruptions were expressed as percentages of households headed by females in an area. Ethnic/racial diversity was derived from a population group and categorized as homogenous or heterogenous. Aside from the explanatory variables, the following co-variables were included in the analyses: place of residence was defined as either rural or urban, and administratively defined by the country. Age of the respondent: 15–19/20–24 years, education attainment (no education, primary, secondary, or higher), and employment status (not working or working). The choice and selection of key explanatory variables and co-variables were informed by their documented significant association with risky sexual practices and other related potential health risks. To make analyses and interpretations simpler and more meaningful, some variables were regrouped from their original categories in the dataset.

**Table 1:** Summary of variables and their categories

Variable type	Variable	Description
Outcome variable	Multiple sexual partnerships	Number of sexual partners in the past 12 months before the survey. This variable was categorized as “1” if the respondent did not have any sexual partner apart from their current sexual partner and “2^+^” if more than one partner
Key explanatory variable	Neighbourhood poverty	Index of community poverty Derived from households without electricity for cooking, heating, and lighting; households without regular refuse collection; households without flush toilets; and households without piped water at the dwelling. Categorized as No or Yes.
	Residential mobility	Percentage of individuals who moved to a municipality in the past five years This variable is categorized as Yes or No
	Family disruption	The measures used for community-level family disruption were female-headed and single-parent-headed. Categorized as male or female
	Ethnic/racial diversity	This was derived from the population group, while ethnic heterogeneity was an index of ethnicity derived from the home language spoken. Categorized as Homogenous and Heterogenous
Co-variables	Place of residence	Rural or urban residence
	Age	Age of respondent. This variable is categorized as 15–19 and 20–24 years
	Education attainment	The highest educational level of respondent. Categorized as Primary or less, secondary and higher
	Employment status	Employment status of the respondent. Categorized as employed or not employed
	Household size	Number of persons in a household. categorized as above and below average

### Statistical analysis

Secondary data analysis from the recent South Africa Demographic and Health Survey was performed using Stata version 17.0 (Stata Corp. Inc. Texas, USA) based on the survey design, the effects of the cluster, and weighting [33]. The Svy command in Stata was used to adjust for the complex survey design of the DHS data. To examine the key demographic and socioeconomic factors, descriptive statistics, which included percentages and frequency were used to assess the relationships between the variables. Cross-tabulation using chi-squared was used to check the association between MSP and selected independent variables. Multivariate results are presented as odd ratios (OR) and adjusted odd ratios (aOR) with 95% confidence intervals (CIs).

A two-level multilevel binary logistic regression model was used to assess the relationship between individual and contextual factors that are associated with MSPs among youths. Using multilevel models allowed for the identification of clustering of the dependent variables at different levels. Random effects in the multilevel modelling were used to show the extent to which MSPs varied between communities (levels higher than the individual). The random effects also showed factors that were omitted from the model or factors that could not be quantified in the survey. Therefore, estimated the degree of correlation in the outcome that existed at the community level, and controls for individual and community factors, thereby acting as residual variation in the outcomes [34]. Two separate multilevel logistic models were fitted for the dependent variable, MSPs. The statistical analysis used in this study fitted because of the homogeneity of the youths in the community and the youths nested within the community, and the dependent variable. This meant that two-level multilevel models were used with youths (level 1) nested within communities (level 2). The model took the following form:

1 $${\rm{Logit}}\,\left( {{{\rm{P}}_{{\rm{ijk}}}}} \right)\,{\rm{ = }}{{\rm{X}}_{{\rm{ijk}}}}{\rm{\beta }}\,{\rm{ + }}\,{{\rm{U}}_{{\rm{jk}}}}{\rm{ + }}\,{{\rm{V}}_{\rm{k}}}{\rm{;}}$$

where P_ijk_ is the probability of having two or more sexual partners for the ith respondent in the jth individual in the kth community. X_ijk_ is a vector of covariates corresponding to the ith respondent in the jth individual in the kth community. Β is a vector of unknown parameters, U_jk_ is the random effect at the individual level, and V_k_ is the random effect at the community level. The variables entered into the model were grouped into individual and community variables. The fitting of the null or empty two-level model, which is referred to as a model with only an intercept and community effects. The equation below was used:

$${\rm{Log}}\,{\rm{(}}\pi ij{\rm{1/}}\pi ij{\rm{)}}\,{\rm{ = }}\beta {\rm{0 + u0j}}$$

The intercept β0 was shared among the communities, whereas the random effect u0j was specific to a particular community j. The random effects were assumed to follow a normal distribution with variance σ^2^uo. Meanwhile, the binary response (RSB) followed the command, which was then followed by a list of fixed-part explanatory variables. This null model contained only an intercept, and no explanatory variables were included. Stata software using the syntax xtmelogit was used to fit the multilevel models for the binary response variables, as shown below using one of the outcome variables:

$${\rm{xtmelogit}}\,{\rm{sexual\_partners}}\,||{\rm{v001:}}\,{\rm{var}}\,{\rm{and}}\,{\rm{xtmelogit}}\,{\rm{v761}}||\,{\rm{v001:}}\,{\rm{var}}$$

Therefore, 8 models were fitted for the dependent variable (MSPs). The first model, the empty model referred to as the ‘null’ model, was fitted without any independent variables. This model included only a random intercept and allowed the observation of the possible presence of a neighbourhood influence on these outcomes. Model 1 (null model) allowed investigation into differences associated with dependent factors in all neighbourhoods. Model 2 is comprised of individual-level characteristics. The aim of the analysis was to determine the degree to which an individual’s attributes demonstrate area-level variations when engaging in MSPs. Model 3 only examined community variables to investigate the influence of community characteristics on each community. Model 4 (full model) included both neighbourhood and individual variables. This model investigated the effects of background characteristics on MSPs and the severity with which community elements modified the correlation between participants and MSPs among young people.

The Variance Partition Coefficient (VPC) was included in this study to assess the degree to which individuals from a community look very similar to others far beyond individuals from other communities regarding the outcome variable (MSPs and inconsistent condom use). VPC was the percentage of the total variance (V_EA_ + V_I_) in the outcome that was attributed to the community/EA level (V_EA_) and was, therefore, a measure of *clustering*.

Model 1 consisted solely of decomposing the total variance of the outcome (VTotal) into its individual (VI) and community/EA (VEA) components, with no explanatory variables. Therefore, in the simple model (model 1), the VPC directly with the intraclass coefficient of correlation, which was a measure of the overall cluster formation of the participant variable of interest in the areas. As a result, the size of this coefficient was critical information in our study because the higher the rate, the more relevant the community level for explaining an individual’s participation in RSB [12]. The intraclass correlation coefficient was defined as the ratio of variance at the neighbourhood level to the total variance (ICC). Because this was a two-level logistic random effects method with a two-intercept variance, the intraclass correlation was: *ρ* = (σ^2^_µ_ / (σ^2^_µ_ + π^2^/3).

Where: p is the intra-class correlation (ICC), σ^2^_µ_ was the variance at the community level _=_ π^2^/3 = 3.29 and represents the level-1 residual variance for a logit model. In conclusion, if most of the variations in each outcome were explained by individual-level measures, the ICC would be close to 0.

### **Ethical consideration**

The study used a secondary dataset from the SADHS with all identifier information removed and the authors sought permission to access the data ICF International website: the DHS Program - Available Datasets). The survey was approved by the Ethics Committee of the ICF Macro at Calverton, USA, and by the National Ethics Committees of South Africa.

## Results

### Profile of the study population

The descriptive statistics are shown in Table 2. A total of (females − 2,621 and males − 1,268) were included in the analysis. The results showed that there were (67.4% females) and (32.6%) males. A larger proportion of females (55.7%) and males (55.5%) were aged 15–19 years. Regarding educational attainment, over two-thirds of the young people had secondary education, but slightly more females (88.0%) than males (82.4%) had secondary education. Most of the female young people (91.4%) were unemployed, compared with 83.7% of male young people. A little over one-half of the female and male young people (53.2%) were urban and rural residents, respectively. Most of the young people (females – 64.4% and males – 52.5%) were found in female-headed households. Approximately 23% of the young people came from areas of high neighbourhood poverty. Most of the female (78.5%) and male (69.4%) young people were found in households with members larger than the average (less than five household members). Considering residential mobility, about 21% of both female and male young people reported having a history of residential mobility. Female and male Black Africans (89.3% and 91.0%, respectively) dominated the sample.

**Table 2:** Description of the study population by gender

	Female	Male
Variables/categories	*N* (%)	*N* (%)
Age (years)		
15–19	1,461(55.7)	704(55.5)
20–24	1,160(44.3)	564(44.5)
**Educational attainment**		
No education	8(0.3)	6(0.5)
Primary	149(5.7)	166(13.1)
Secondary	2,306(88.0)	1,045(82.4)
Higher	158(6.0)	51(4.0)
**Employment status**		
Not working	2,396(91.4)	1,061(83.7)
Working	225(8.6)	207(16.3)
**Place of residence**		
Rural	1,228(46.8)	674(53.2)
Urban	1,393(53.2)	594(46.9)
**Female-headed households**		
No	932(35.6)	602(47.5)
Yes	1,689(64.4)	666(52.5)
**Neighbourhood poverty**		
No	2,025(77.3)	979(77.2)
Yes	596(22.7)	289(22.8)
**Number of household members**		
≤ average	564(21.5)	388(30.6)
> average	2,057(78.5)	880(69.4)
**Residential mobility/instability**		
No	2,071(79.0)	997(78.6)
Yes	550(21.0)	271(21.4)
**Ethnicity/racial diversity**		
Black/African	2,340(89.3)	1,154(91.0)
White	33(1.3)	20(1.6)
Coloured	230(8.8)	84(6.6)
Indian/Asian	18(0.7)	10(0.8)

### Bivariate analysis

The bivariate relationship between the MSP individual-level factors of female and male young people is presented in Table 3. The results revealed that the individual and community level factors were significantly associated with MSP, except for the place of residence and neighbourhood poverty. Concerning individual-level factors of MSP, the results showed that a higher percentage of MSP was found among female (79.2%) and male (84.4%) young people aged 20–24. The results further revealed that female and male young people with primary or less education had the lowest percentage of MSP (41.4% and 38.4%, respectively), whereas those with higher education had the highest percentage of MSP (73.4% and 94.1%, respectively). In addition, female (78.2%) and male (83.6%) young people who were employed had a higher percentage of MSP. Considering community-level factors and MSP, the results significantly showed that a higher percentage of MSP was reported among female and male young people (59.4% and 58.3%, respectively) from female-headed households; females and males (60.5% and 70.4%, respectively) from households with a household size was smaller than or equal to the average of 3 members, as well as females and males (61.5% and 73.4%, respectively) with a history of residential mobility. The results further revealed that the highest percentage of MSP was reported among female (58.3%) and male (63.6%) Black Africans in the sample.

**Table 3:** Variations in the association of multiple sexual partnerships with individual-level, household-level, and community-level factors

	Female		Male	
**Variables/categories**	N (%)	Chi-square	N (%)	Chi-square
**Age (years)**		453.8***		220.6***
15–19	550(37.7)		307(43.6)	
20–24	919(79.2)		476(84.4)	
**Educational attainment**		33.1***		64.7***
Primary or less education	65(41.4)		66(38.4)	
Secondary	1,288(55.9)		669(64.0)	
Higher	116(73.4)		48(94.1)	
**Employment status**		49.1***		49.9***
Not working	1,293(54.0)		610(57.5)	
Working	176(78.2)		173(83.6)	
**Place of residence**		1.5		0.7
Urban	765(54.9)		374(47.8)	
Rural	704(57.3)		409(60.7)	
**Female-headed households**		22.2***		7.2**
No	465(49.9)		395(65.6)	
Yes	1,004(59.4)		388(58.3)	
**Neighbourhood poverty**		8.4*		0.2*
No	1,133(56.0)		603(61.6)	
Yes	336(56.4)		180(61.8)	
**Number of household members**		5.7*		17.5***
≤ average	341(60.5)		273(70.4)	
> average	1,128(54.8)		510(58.0)	
**Residential mobility/instability**		8.3**		19.9***
No	1,131(54.6)		584(58.6)	
Yes	338(61.5)		199(73.4)	
**Ethnicity/racial diversity**		46.9***		20.4***
Black/African	1,363(58.3)		734(63.6)	
White	11(33.3)		7(35.0)	
Coloured	92(40.0)		39(46.4)	
Indian/Asian	3(16.7)		3(30.0)	

Note: *p < 0.05; **p < 0.01; ***p < 0.001

### Multilevel models

The results presented in Table 4 show fixed-effect (measures of association) and random-effect (measures of variation) results from multilevel analysis with MSP as the outcome variable and that there is considerable between-community heterogeneity. For instance, for the MSP as shown in the community level variance, female young people (τ = 0.17, *p* < 0.001) and male young people (τ = 0.55, *p* < 0.0001) respectively in the empty model were significant, indicating differences across the clusters where young people lived. In addition, the intraclass correlation coefficient (ICC) in the empty model for MSP showed that 0.5% and 14% of the variance in the log odds of reporting MSP could be attributed to the community level. Variations across community clusters remained statistically significant even after controlling for individual-level and community-level factors, thereby supporting for the use of multilevel modelling to account for community variations.

Table 4 also presents the results of the fixed effect of the unadjusted results of the associations between MSP and individual-level and community-level factors. For individual-level factors, the results showed that age was significantly associated with MSP for both female and male young people. For instance, the odds of young females and males aged (20–24 years) had significantly increased odds of having MSP (OR = 6.32, 95% CI: 5.29–7.54 and OR = 6.99, 95% CI: 5.33–9.18, respectively), compared with those (15–19). Considering educational attainment, the odds of having MSP were 3.6 and 1.6 times higher among female and male young people who had attained a higher education, compared with those with primary or less education. Surprisingly, being employed significantly increased the odds of having MSP for both females (OR = 3.06; 95% CI: 2.21–4.24) and males’ young people (OR = 3.76; 95% CI: 2.55–5.54).

At the community-level measures, the odds of engaging in MSP significantly increased among females (OR = 1.47; 95% CI: 1.25–1.73) but decreased among males (OR = 0.73; 95% CI: 0.58–0.92) from female-headed households (family disruption). The odds of having MSP significantly decreased with an increase in the average number of households among females (OR = 0.79; 95% CI: 0.66–0.96) and males (OR = 0.58; 95% CI: 0.45–0.75) young people. In addition, female, and male young people with a history of residential mobility/instability (OR = 1.33; 95% CI: 1.09–1.61 and OR = 1.95; 95% CI: 1.45–2.63) were more likely to have reported MSP. Concerning ethnicity/race, the results showed that both gender who reported to be Coloured, Whites and Indians/Asians were less likely to have MSP than to their Black African counterparts.

The adjusted logistic regression results of the associations between MSP and individual and community-level factors, as presented in Table 4, showed that older female and male young people were more likely to report having MSP. For instance, the odds of having MSP significantly increased among female and male young people aged 20–24 (aOR = 5.82, 95% CI: 4.82–7.03 and aOR = 5.43, 95% CI: 4.02–7.35, respectively), compared with those aged 15–19. As observed in the unadjusted analysis, the odds of having MSP increased among female and male young people with additional educational attainment. Female and male young people with secondary education had higher odds of having MSP (aOR = 1.52, 95% CI: 1.06–2.18 and aOR = 2.51, 95% CI: 1.74–3.61, respectively), relative to those with primary or less education. A similar significant result of increased odds of having MSP was observed among male young people with higher education (aOR = 8.05, 95% CI: 2.31–28.11). Additionally, being employed was found not to be a protective factor for engaging in MSP. The results showed that having MSP significantly increased among employed females (aOR = 1.65; 95% CI: 1.14–2.38) and males (aOR = 1.88; 95% CI: 1.19–2.96). The odds of having MSP were significantly reduced among female and male young people who reported to be Coloured, White and Indians/Asians, relative to their Black African counterparts.

**Table 4:** Measures of variations and factors associated with multiple sexual partnerships identified by multilevel logistics models

	Female			Male		
Variables/categories	*N*	OR (95% CI)	aOR (95% CI)	*N*	OR (95% CI)	aOR (95% CI)
Fixed Effects Individual variables Age 15–19 (RC) 20–24	550 919	1.00 6.32(5.297.54) ***	1.00 5.82(4.82–7.03) ***	307 476	1.00 6.99(5.33–9.18) ***	1.00 5.43(4.02–7.35) ***
**Educational level** Primary or less (RC) Secondary Higher	65 1288 116	1.00 1.79(1.292.49) *** 3.91(2.436.28) ***	1.00 1.52(1.06–2.18) * 1.52(0.89–2.58)	66 669 48	1.00 2.86(2.05–3.98) *** 25.7(7.69–85.8) ***	1.00 2.51(1.74–3.61) *** 8.05(2.31–28.11) **
**Employment status** Not working (RC) Working	1293 176	1.00 3.06(2.214.24) ***	1.00 1.65(1.14–2.238) **	610 173	1.00 3.76(2.55–5.54) ***	1.00 1.88(1.19–2.96) **
**Community Variables**						
**Neighbourhood poverty** No Yes	1133	1.00 1.47(1.25–2.38) **	- -	603 180	1.00 1.03(0.79–1.35)	- -
**Residential mobility** No Yes	1131	1.00 1.33(1.09–1.61)	1.00 1.11(0.89–1.38)	584 199	1.00 1.95(1.45–2.63) ***	1.00 1.31(0.93–1.85)
**Place of residence** Urban Rural	765 704	1.00 0.91(0.78–1.06)	- -	374 409	1.00 1.10(0.89–1.39)	- -
**Female-Headed household** No Yes	465 100	1.00 1.47(1.25–1.73) ***	1.00 1.19(0.99–1.43)	395 388	1.00 0.73(0.58–0.92) **	1.00 0.89(0.68–1.17)
**Number of household members** < Average > Average	341 112	1.00 0.79(0.66–0.96) *	1.00 1.04(0.83–1.29)	273 510	1.00 0.58(0.45–0.75) ***	1.00 0.79(0.59–1.06)
**Ethnicity/Racial diversity** Black/African hite Coloured Indian/Asian	1363 11 92	1.00 0.36(0.17–0.74) ** 0.48(0.36–0.63) *** 0.14(0.04–0.50) **	1.00 0.27(0.12–0.60) ** 0.50(0.36–0.68) *** 0.10(0.03–0.37) **	734 7 39 3	1.00 0.31(0.12–0.78) * 0.50(0.32–0.77) ** 0.25(0.06–0.95) *	1.00 0.2790.09–0.78) * 0.28(0.160.48) *** 0.12(0.03–0.57) **
**Random Effects** Community level variance Residual intra-class corr. Log likelihood AIC		0.17(0.08–0.37) ** 0.05 -1792.84 3589.68		0.55(0.27–1.10) *** 0.14 -827.96 -1659.92		

Note: *P < 0.05; **P < 0.01; ***P < 0.001; RC = Reference category

## Discussion

The objective of this study was to examine the contextual factors associated with MSPs among young people in South Africa, with an emphasis on the role of community-level social disorganisation. The study found that individual and contextual-level determinants of social disorganisation are important predictors of MSPs among young people in South Africa. Therefore, gender disparities were observed in multiple sexual partnering among young people in South Africa. For instance, male youth were found to be more likely to engage in MSPs than their female counterparts, although, with a slightly different. This finding agrees with previous observations that showed that males tend to indulge in risky sexual behaviour, including having a greater number of sex partners than their female counterparts [35–38].

Our study revealed that at the individual level, age was found not to be a protective factor for having MSPs for both female and male young people. In line with Khan’s [39] observation, our result could be attributed to the fact that older female and male young people tend to have more confidence, as well as better knowledge and experience, which might influence their engagement in MSPs. Surprisingly, the findings of this study established that increased education was associated with MSPs among female and male young people. This corroborates previous findings that the growing level of female and male young people’s exposure through education could influence their sexual behaviour, which includes having MSPs [40, 41]. This is reflected in the role education plays in societal transformation and the provision of information for the knowledge of healthy sexual behaviour. This is mostly achieved through sexual and reproductive health education that most young people receive in the country, especially from academic institutions. Employment status also played a role in youth engagement with MSPs. For instance, the results showed that being employed significantly increased the likelihood of engaging in MSP for both females and male young people. These findings support of recent observations that suggest employed female and male young people tend to have MSPs, because of their financial security, which gives them false hope to indulge in risky sexual behaviour [42]. Nevertheless, money predisposes young people to engage in risky sexual behaviour such as MSPs, especially when they feel that the situation could financially be handled.

This study has established that the association between MSPs and contextual factors has been reflected in many previous studies [16, 17, 20]. Family support is of special importance to young people, especially at the community-level in many provinces in South Africa, where youth congregate. It was found that youths from female-headed households tended to engage in MSPs, unlike those from intact families (both father and mother). Therefore, family disruption played a key role in STIs prevention among young people, especially females, who might not be closely monitored by their mothers, unlike their male counterparts. This is because there is a tendency for female young people to meet with their sexual partners outside their homes, compared to males who most often invite their sexual partners to the family house. Furthermore, previous studies have already documented how coming from a disrupted family affects the sexual behaviours of young people [43]. Girls are expected to be protected by both parents, which can become difficult in a single parent’s household. Therefore, practical support, as well as emotional support, from both parents (father and mother) can be a protective factor for the youth to engage in risky sexual behaviour, hence, it enhances their self-esteem, self-confidence, and self-autonomy in sexual relationships [44].

The study has shown that poverty affects the health outcomes of young people in South Africa. In line with the observations of previous studies in South Africa, the results established that residing in areas of high poverty is a risk factor for risky sexual behaviours among youths, such as MSPs in South Africa [12]. The reason for this could be that members of poor households are generally exposed to high levels of financial incapacitation, which increases the risk of MSP, which affects the health and well-being of youths [14]. The results suggest that interventions aimed at improving youths’ SRH should consider the roles of neighbourhood poverty within the community.

Our study revealed that household size has been well established in the literature as an important determinant of youth. Household size was significantly associated with MSP and was identified as the most important family-level determinant of MSP among youths in this study. However, coming from a household larger than the average size reduced the likelihood of MSP among youths. Govender et al. [10], found that household size was significantly associated with MSP in South Africa. For instance, this study revealed that 60.5% and 70.4% of female and male youths from household size number, which was smaller than or equal to the average of 3 members, were found to engage in MSPs. Adolescents and young adults from a larger family size tend to undergo social, emotional, and physical isolation and pressure in their communities, which exposes them to engaging in MSPs, although, this may be due to smaller sample size of this study. This is heightened because of breakdown of social norms existing in such communities, where youth activities are no longer monitored. Further studies with larger sample sizes, especially longitudinal data, are required to determine the trends in MSP practises among youths and to improve the power of subgroup analyses.

This study established that residential mobility as a perceived risk factor for youth engagement in MSPs has not been widely investigated in South Africa, using a national survey. In Nigeria, a residential mobility study [45] found that having a history of residential mobility/instability significantly increased the practice of MSP among young people. Similarly, a study of residential mobility in Asia [46] found that residential mobility comes with challenges of cultural norms and values that encourage young people to engage in MSPs. These findings are also reflected in our study, whereby youth who have a history of movement from one location to the other regularly tend to engage in MSPs. This again reflects the importance of cultural differences and assimilation in decision making and power dynamics in sexual behaviour, whereby male’s decision preferences in sexual matters considered to be more important. Consequently, change of residence among female and male young people could heighten their indulgence in MSPs, thereby increasing HIV acquisition risks [47, 48]. This finding demonstrates the need for more knowledge on sexual health and reproductive rights of young people, particularly female youths.

There were differences in multiple sexual partnering with ethnicity, as we observed that coming from other African countries with diverse contradictory cultural norms and values regarding sexual behaviour might lead to MSPs. This calls for urgent policy concerns in line with previous observations regarding youths having contradictory cultural norms and values about healthy sexual behaviour because of social change [49, 50]. The findings suggest that the interplay between ethnic diversity and MSPs underlines the influence of cultural norms and values modified by globalisation in shaping sexual behaviour and its consequences for MSP engagement. Thus, young people’s behaviours naturally reflect specific cultural or national traditions peculiar to different ethnic backgrounds or identities, hence, practices become diverse in the same way.

This study is not without some limitations. The present analyses were limited to never-married young people in South Africa. This restriction to never-married young people, without the views of married youth or those who did not respond to the questions, could have a strong potential for reporting bias/discordance regarding social disorganisation measures influencing MSP. Despite these limitations, the findings are important for more strategic policies and programmes in preventing sexually transmitted infections, including HIV, and monitoring intervention programmes to control the spread of the epidemic, especially among never-married young people in South Africa.

## Conclusions

In South Africa, contextual factors influence adolescents to engage in multiple sexual partnerships. Neighbourhood poverty, residential mobility, family dysfunction, and ethnic/racial dynamics were the major determinants of multiple sexual partnerships among young people. Promoting unbiased comprehensive sexual education at the community level would be key to reducing the high prevalence of risky sexual behaviour. The findings from this study, would inform and help policymakers to consider appropriate policy options to prevent the high rate of future HIV/AIDS cases in South Africa. We recommend that future studies should investigate other contextual factors, such as community social processes: social norms, social networks, social relations, and social control, which may account for the unexplained community-level predictions of multiple sexual partnerships among young people in South Africa.

## Data Availability

South Africa Demographic and Health Survey (2016) datasets are freely available at https://www.dhsprogram.com/data/dataset.

## Abbreviations

AIDS: Acquired Immunodeficiency Virus
aOR: Adjusted Odds Ratio
DHS: Demographic and Health Survey
HIV: Human Immunodeficiency Virus
MSP: Multiple Sexual partnerships
NDoH: National Department of Health
OR: Unadjusted Odds Ratio
The RC: Reference category
SADHS: South African Demographic and Health Survey
SAMRC: South African Medical Research Council
SDT: Social Disorganisation Theory
Stats SA: Statistics South Africa
STIs: Sexually Transmitted Infections
UNAIDS: Joint United Nations Programme on HIV and AIDS
WHO: World Health Organisation
